# Efficacy of MAGE-A4 long peptide as a universal immunoprevention cancer vaccine

**DOI:** 10.1186/s12935-024-03421-2

**Published:** 2024-07-03

**Authors:** Lanqi Cen, Zhe Zhang, Yi Sun, Nandie Wu, Jie Shao, Zhaoye Qian, Manman Tian, Yaohua Ke, Baorui Liu

**Affiliations:** 1 Department of Oncology, China Pharmaceutical University Nanjing Drum Tower Hospital, Nanjing, 210000 China; 2 Department of Oncology, Affiliated Hospital of Medical School, Nanjing Drum Tower Hospital, Nanjing University, Nanjing, 210000 China; 3 Center of Clinical Oncology, The Affiliated Hospital of Xuzhou Medical University, Xuzhou, 221000 China; 4 Department of Oncology, Nanjing Drum Tower Hospital Clinical College of Nanjing Medical University, Nanjing, 210000 China

**Keywords:** Cancer peptide vaccine, Long peptide, MAEG-A4, R848, Immunotherapy

## Abstract

**Background:**

The clinical application of peptide vaccines in tumor immunotherapy holds significant promise. Peptide-based tumor vaccines are currently subject to certain limitations in clinical trials, including the challenge of inducing a sustained response from CD4^+^ T helper cells and cytotoxic T lymphocytes (CTL), as well as human leukocyte antigen (HLA) restrictions.

**Methods:**

Through the utilization of biological information methodology, a screening process was conducted to identify three potential long peptides that are specifically targeted by the MAGE-A4 antigen. The candidate long peptides were subjected to in vitro testing using human peripheral blood lymphocytes as samples to evaluate their immunogenicity and immune function. The antitumor properties and preliminary mechanism of the long peptide vaccine were investigated through the use of a mouse model designed for the prevention of triple negative breast cancer (TNBC).

**Results:**

Three predicted multi-epitope long peptides targeting MAGE-A4 have shown to have a strong immunogenicity, with a total positive rate of 72% across different HLA subtypes in Chinese populations. they can also increase the levels of the costimulatory factor CD137 and tumor necrosis factor-alpha (TNF-α), activate T cells, and boost the cytotoxic activity. Results from an animal study have revealed that the long-peptide vaccine, both on its own and in combination with R848, has displayed impressive anti-tumor and target-specific capabilities. Moreover, it has the ability to increase the expression of effector memory T cells and central memory T cells.

**Conclusions:**

This study was the first to screen three multi-epitope long peptides targeting MAGE-A4 and assess their immunogenicity, immune function, and potential as adjuvant peptides. The results showed that the MAGE-A4 long peptide vaccine can be used as a novel immunoprophylaxis method to prevent TNBC. Moreover, the proposed development model is capable of screening multiple target antigens, which lead to its clinical application.

**Supplementary Information:**

The online version contains supplementary material available at 10.1186/s12935-024-03421-2.

## Introduction

The predicament of tumor therapy has been substantially alleviated by the implementation of immunotherapy strategies [1]. Peptide-based tumor vaccines have emerged as a promising area of research in the field of tumor immunotherapy. The primary hindrances of clinical trials involving short peptide-based tumor vaccines are associated with immune tolerance. In comparison to short peptide vaccines, multi-epitope tumor peptide must be internalized and processed by antigen-presenting cells (APCs) prior to presentation [2]. Professional APCs can process pools of multi-epitope peptides, and they are adept at extracting multiple HLA class I and II peptide epitopes for display on the cell surface. Multi-epitope tumor peptide vaccines have the ability to target a broader spectrum of tumor antigens, thereby eliciting a more targeted immune cell response, this strategy is aimed at addressing tumor heterogeneity and circumventing immune escape [3]. Due to reduced major histocompatibility complex (MHC) restriction, this approach can be more broadly utilized across diverse populations. Moreover, both cytotoxic CD8^+^T cells and helper CD4^+^T cells are stimulated to generate a sustained immune response [4–6]. A multitude of clinical trials have been conducted to appraise the safety and efficacy of multi-epitope tumor peptide vaccines that aim to target Survivin, NY-ESO-1, WT-1, IDO, PD-L1, and neoantigens [3, 7–11].

The protein known as MAGE-A4, a cancer-testis antigen, is typically expressed in the early stages of embryonic development and in immune-privileged areas of healthy adult tissue [12]. However, its expression becomes high expression in several types of solid tumors, including melanoma, triple-negative breast cancer, non-small cell lung cancer, esophagus cancer, which provides immunotherapy opportunity [13–18]. As mentioned, multi-epitope long peptide vaccines have more advantages than short peptide vaccines, so we are aiming to create a comprehensive multi-epitope long peptide vaccine that is focused on MAGE-A4.

In clinical trials, tumor peptide vaccines and adjuvants are widely utilized as recognized strategies to improve vaccine efficacy. Adjuvants commonly employed include complete Freund’s adjuvant, incomplete Freund’s adjuvant (IFAs), toll-like receptor (TLR) agonists, and cytokines [19]. TLR agonists have garnered significant attention due to their safety and efficacy. R848, a TLR agonist, has been extensively employed as an adjuvant in vaccine clinical trials for the management of melanoma and other solid tumors [20, 21]. In comparison to CpG, another TLR agonist, R848 is a more efficacious option, with a lower production cost and a flexible and optimized structure [22]. It is a vaccine adjuvant with immense potential for transformation.

In light of the aforementioned foundation, a bioinformatics approach was utilized to screen three potential long peptides targeting MAGE-A4, which were subsequently combined with TLR adjuvant R848 to explore their immune effects in animal and human peripheral blood mononuclear cells. The findings indicate that the long peptide vaccine exhibited the ability to impede tumor growth and extend survival, while also demonstrating robust immunogenicity and immune function in 25 human blood samples. The proposed model of development has the capacity to screen multiple target antigens with efficacy, offering a fresh approach and methodology for the evolution of clinical tumor vaccines. Additionally, it possesses the potential for eventual clinical translation.

## Materials and methods

### Universal T-cell epitope design, prediction and peptide synthesis

A formal survey was carried out on the HLA class I and HLA class II restrictive locus, covering 812,211 individuals throughout 31 provinces, autonomous regions, and municipalities in China [23]. We have opted to choose six HLA-A alleles and eight HLA-DRB1 alleles, which have been chosen to cover a broad range of the Chinese population. The FASTA format amino acid sequences of human MAGE-A4, identified by accession number WBY73526.1, were obtained from the NCBI database (https://www.ncbi.nlm.nih.gov/). The TMHMM tool was utilized to exhibit whether the peptides are situated within transmembrane regions or not (http://www.cbs.dtu.dk/services/TMHMM/). The identification of candidate epitopes for MHC class I was carried out using a combination of methodologies, including IEDB (http://tools.iedb.org/mhci/), SYFPEITHI (http://www.syfpeithi.de/0-home.htm), and NetMHCpan EL 4.0 (http://www.cbs.dtu.dk/services/NetMHCpan/). Similarly, for MHC class II, the IEDB (http://tools.iedb.org/mhcii/) recommended binding prediction tool was employed.

A number of epitopes exhibiting a high level of affinity with multiple MHC Class I and Class II alleles were deemed as probable epitopes. The screening of potential epitopes was based on a set of criteria, which included strong binding for those with an affinity rank value of less than 0.5% binding with various MHC Class I molecules. Conversely, epitopes with a rank value greater than 0.5% but less than 2% were considered to have weak binding. Our approach to selecting candidate peptides involved prioritizing those that displayed robust affinity and high scores across multiple prediction software, as determined through our comprehensive evaluation process. Owing to the relatively greater degree of promiscuity in peptide binding to MHC Class II molecules, the level of accuracy in predicting MHC-II binding by the algorithm is lower than that of the algorithm used for predicting MHC-I binding. Therefore, it is imperative to choose a greater number of MHC-II categories. Only epitopes that display an affinity rank value below 10% for binding to specific MHC-II molecules have been selected. The sequences exhibiting a strong binding potential towards the specified HLA typing and encompassing a wide spectrum of HLA loci are integrated. According to the rules, MLP-1 (MAGE-A4_102 − 130_, AESLFREALSNKVDELAHFLLR.

KYRAKEL), MLP-2 (MAGE-A4_134 − 160_, AEMLERVIKNYKRCFPVIFGKASESLK), and MLP-3 (MAGE-A4_274 − 306_, RALAETSYVKVLEHVVRVNARVRIAYPSLR) have been projected as the three long peptides of MAGE-A4(MLP). The solubility of the potential multi-epitope peptide in water was evaluated through the utilization of Pepcalc (http://pepcalc.com/). The synthesis and validation of three candidate long peptides, HIV irrelevant peptide (YVDRFYKTLRAEQASQEV), MAGE-A4_283 − 291_ (SYVKVLEHV) were performed by GenScript (Nanjing, China).

### Human peripheral blood mononuclear samples from healthy individuals

The extraction of peripheral blood mononuclear cells (PBMCs) was carried out using blood samples from 25 healthy donors. All human blood samples experiments were performed in accordance with the Ethics Committee of Drum Tower Hospital (2020-203-02) and in accordance with the Declaration of Helsinki, International Conference on Harmonization/GCP guidelines. The detection of Human Leucocyte Antigen (HLA) from these samples was accomplished through the utilization of the HLA high resolution sequencing typing system, which was developed by Beijing Boao Jingdian Biotechnology Co LTD. According to the protocols outlined by GE healthcare, PBMCs were isolated through the employment of the Ficoll/Hypaque density-gradient centrifugation technique. Briefly, an equal volume of Ficoll reagent was slowly added to a centrifuge tube containing 10mL of blood sample, followed by centrifugation at 800 g for 25 min. A second ring-shaped milky lymphocyte layer was aspirated, 10 to 20mL of PBS was added, and the cells were mixed. The samples were then centrifuged at 300 g for 10 min. After repeated washing for 2 times, PBMCS were finally obtained. The cells were subsequently cryopreserved with a solution of fetal bovine serum supplemented with 10% dimethyl sulfoxide (DMSO, Aladdin, China) and then stored in a liquid nitrogen environment.

### Animals and cell lines

Female Balb/c mice, aged 5–6 weeks, were obtained from the Shanghai Sippr-BK laboratory animal Co. Ltd. in Shanghai, China. All animal experiments referring to animals were performed in accordance with the Guide and Care and Use of Laboratory Animals from the National Institutes of Health and ARRIVE. The animal testing was carried out in adherence to the guidelines laid down by the Laboratory Animal Care and Use Committee of the Affiliated Nanjing Drum Tower Hospital of Nanjing University Medical School (2021AE01084). The Cell Bank of the Chinese Academy of Sciences in Shanghai, China was the source of procurement for the Murine breast cancer cell (4T1), A375 is a human melanoma cell line that expresses MAGE-A4 and its HLA typing is HLA-A *02:01, HLA-A *01:01. NUGC-4 is a human gastric cancer cell line and its HLA typing is HLA-A *24:02, but it doesn’t express MAGE-A4. We plused the NUGC-4 cells with a short peptide (MAGE-A4_283 − 291_: SYVKVLEHV) that was included in the MLP-3 amino acid sequence, this short peptide sequence was predicted to have a high affinity with HLA*24:02, thus MAGE-A4_283 − 291_-pulsed NUGC-4 can be used as a target cell [24, 25].

The establishment of 4T1-MAGE-A4^+^ cells involved the transfection of the whole human MAGE-A4 overexpression vector into 4T1 cells, which was executed by BrainVTACo. Ltd (China). Negative control cells were utilized, which lacked exogenous genes. The femur and tibia of Balb/c mice were utilized to collect bone marrow mesenchymal hepatocytes, which were subsequently cultured with a medium consisting of 90% RPMI 1640, 10% FBS, and 1% P/S at 37 ℃ in 5% CO2. The cells were then induced to differentiate into DCs by adding rmIL-4 (10ng/mL, Pepro Tech, USA) and rmGM-CSF (20ng/mL, Amoytop, China). Every 48 h, the medium was renewed during the cultivation period, and all the supernatant was discarded, with all the adherent cells being harvested as immature BMDCs (bone Marrow-Derived Dendritic Cells) on the 8th day [26].

### Peptide-induced antigen-specific cytotoxic T-lymphocyte assay

According to the method of cytotoxic T lymphocyte (CTL) culture previously reported [27]. Briefly, the initial step in the experimental procedure involved the thawing of peripheral blood mononuclear cells (PBMCs), followed by an overnight rest period in a medium containing 90% AIMV (Invitrogen Gibco, USA), 0.5% FBS (Invitrogen Gibco, USA), rmGM-CSF (40 ng/mL, Amoytop, China), and IL-4 (20 ng/mL, Pepro Tech, USA). Subsequently, the cells were seeded into 96-well plates at a density of 6 × 10^5^ cells per well, with a volume of 100 µL per well. On the day1, every candidate multi-epitope peptide was introduced into each well at a concentration of 50 µg/mL. Subsequently, after a time lapse of 4 and 4.5 h, resiquimod (R848, 3 µg/mL, MedChemExpress, China) and LPS (50 ng/mL, Merck, USA) were added to the wells, respectively. On the day2, all the cells were amassed and re-suspended in a medium that consisted of 90% AIMV, 10% FBS, and 1% P/S, with the inclusion of IL-2 (24 IU/mL, Pepro Tech, USA) and IL-7 (50 ng/mL, Pepro Tech, USA). The medium was substituted halfway every two days until the 14th day. Induced cells were used in T-cell cytotoxicity assay. On the 13th day, following the procedures outlined for preparing fresh peptide-pulsed cells during the initial two days, fresh cells were obtained. On the 14th day, these newly obtained cells, which had been pulsed with peptides, were mixed with the previously obtained cells and co-cultured in AIMV, the ratio of cells was 2:1. The resulting cells were then utilized in IFN-γ ELISpot assays, as well as intracellular and extracellular cytokine staining. The resulting cells were then utilized in Interferon γ (IFN-γ) ELISpot assays, as well as intracellular and extracellular cytokine staining. The immunogenicity of MLP was confirmed by inducing splenocytes from Balb/c mice using the same induction method as described previously.

### IFN-γ ELISpot assay

In order to establish the frequency of T cells that secrete IFN-γ, previously induced cells (2 × 10^5^/well) and newly pulsed cells with peptides (1 × 10^5^/well) were placed on 96-well plates that had been pre-coated with IFN-γ ELISpot for a duration of 18–20 h (*n* = 3). In short, after lysing the cells and washing the plates, the diluted detection antibody IFN-γ (1:100 dilution and 100 µL per well) was added to the plates. The plates were then incubated for one hour at a temperature of 37℃. Then the plates were washed again, the diluted streptavidin-HRP (1:100 dilutions, 100 µL/per well) was added and incubated at 37℃ for another 1 h. The wells were treated with a mixture of 3-Amino-9-ethylcarbazole (AEC) solution, and subsequently, the plates were placed in a dark room at room temperature. The development process was monitored at 5-minute intervals, and the reaction was halted by adding deionized water. The detection was performed by human IFN-γ ELISpot kit (Dakewe, China.) operation manual. Plates were scanned by ELISPOT CTL Reader (Cellular Technology Inc., USA) and the results were analyzed with Elispot software (AID Diagnostika GmbH, Germany). The frequency of IFN-γ-secreting T cells method in mice is the same as above, the detection was performed by mouse IFN-γ ELISpot kit operation manual.

### Cytokine assay

The objective of this experiment was to analyze the secretion of cytokines both intracellularly and extracellularly. To achieve this, the cells that were previously induced (2 × 10^5^/well) and the newly peptide-pulsed cells (1 × 10^5^/well) were co-cultured on 96-well plates for a duration of 18 h. The following monoclonal antibodies were used for flow cytometry and purchased from Biolegend: CD3-FITC, CD4-PerCP-Cy™5.5, CD8-PerCP-Cy™5.5, CD8-APC, CD137-PE, TNF-α-APC. The detection was performed by Fixation/Permeabilization Solution Kit (BD Biosciences, USA). Finally, the flow data were subsequently acquired with CytoFLEX (Beckman coulter Inc., USA) and analyzed using the FlowJo software.

### Cytotoxicity assay

The evaluation of cultured CTLs’ cytotoxicity against A375 cells and MAGE-A4_283 − 291_ peptide-pulsed NUGC-4 was carried out using the CFSE/PI assay. T cells induced by the corresponding peptide were used as effector cells, A375 cells and MAGE-A4_283 − 291_ peptide-plused NUGC-4 cells were served as target cells, T cells that were not induced by peptide served as controls. Various effector-to-target (E: T) ratios (5:1, 10:1, 20:1) were employed to co-culture CFSE (Abcam Plc, UK) labeled target cells (2 × 10^4^ /well) with effector cells in 96-well plates. After 8 h, the mixed cells were collected and incubated with Propidium iodide (PI) (100 ng/mL, Merck, Germany) for 5 min in dark at room temperature. Flow data were acquired with CytoFLEX and analyzed by FlowJo software.

### In vitro BMDCs stimulation

To determine the stimulatory impact of the combination of MLP and R848 on DCs (dendritic cells) in vitro, immature DCs were collected and suspended in a cytokine-free complete medium. These cells were then seeded in a 96-well plate at a density of 1 × 10^4^ cells per well and co-cultured with R848 (3 µg/mL), MLP (50 µg/peptide), or MLP + R848 (50 µg/peptide fused with 3 µg/mL R848) for a period of 24 h. Following this, the cells were amassed and incubated with anti-FITC-CD11c monoclonal antibody, anti-PE-CD86 monoclonal antibody, and anti-APC-CD80 monoclonal antibody (Biolegend, USA) for a period of 30 min in a dark environment at a temperature of 4℃. Flow data were acquired with CytoFLEX and analyzed by FlowJo software.

### Animal experiments

For immunogenicity studies of MLP peptide vaccine in vivo. Balb/c mice were subjected to immunization with various groups, including PBS (50 µL/mouse), HIV (60 µg/mouse), R848 (10 µg/mouse), MLP (total weight 180 µg, 60 µg of each of the 3 long peptides within the mixture/mouse), and MLP + R848 (a fusion of MLP, 10 µg R848/mouse). This was done via subcutaneous injection of a 50 µL volume on day 21, 18, 16, 14and 7 prior to tumor cell implantation. The left and right abdominal regions of Balb/c mice were subcutaneously inoculated with 3 × 10^5^ 4T1-MAGE-A4^+^ cells and 3 × 10^5^ 4T1-MAGE-A4^−^ cells, respectively, 7 days after the last vaccine was administered. Tumor volume was measured unblinded with a caliper, the tumor sizes and body weights were measured once every two or three days. To identify the immune response triggered by the vaccine containing MLP in vivo, we replicated the previous experiment and euthanized all the Balb/c mice after 28 days of the initial vaccine administration. Subsequently, we extracted the lymph nodes and spleens for further analysis. The following monoclonal antibodies were used for flow cytometry and purchased from Biolegend: CD4-PC7, CD8-PerCP-Cy™5.5, CD62L-APC, CD44-PE. Flow data were acquired with CytoFLEX and analyzed by FlowJo software.

The sacrifice of all Balb/c mice was carried out on day 7 after the last administration. Following this, a dissection of the organs, namely the heart, liver, spleen, lung, and kidney, was carried out, after which they were treated with a fixative containing 4% formaldehyde. Paraffin-embedded slides, with a thickness of 4 μm, were meticulously prepared and subjected to staining with hematoxylin and eosin (H&E) for the purpose of histological analysis under an optical microscope (Leica, Germany). the levels of aspartate aminotransferase (AST), alanine aminotransferase (ALT), alkaline phosphatase (ALP), blood urine nitrogen (BUN), and creatinine (CREA) among the five groups in serum were detected.

### Statistical analysis

The values displayed were expressed as the mean ± SEM for at least three independent experiments. The performance of statistical analysis and chart creation was accomplished through the utilization of Graphpad Prism 8.0. The comparison between two groups was conducted using the Unpaired Student’s t test, while multiple comparisons were carried out using one-way analysis of variance (ANOVA) with Bonferroni correction. The log-rank (Mantel-Cox) test was applied as the means of survival analysis.

## Results

### MAGE-A4 sequence analyze, structural and T-cell multi-epitopes prediction

**Fig. 1:**
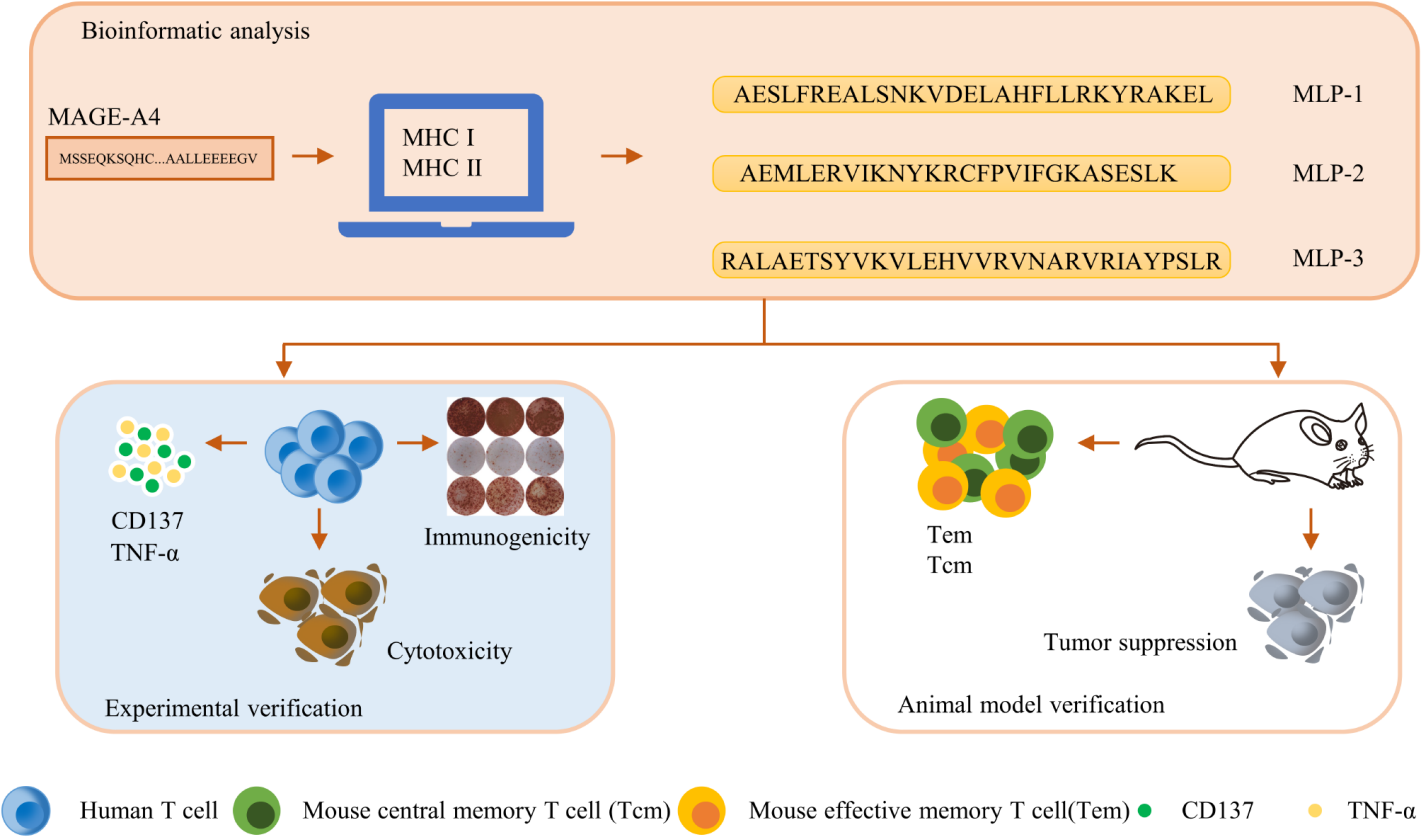
Schematic diagram of the screening procedure for the MAGE-A4 long peptides(MLPs)

Our aim is to produce an immunogenic cancer vaccine that would be both universal and efficacious, the screening process is shown in Fig. 1. To achieve this, we have designed the MLP vaccine, which consists of MAGE-A4_102 − 130_ (MLP-1, AESLFREALSNKVDELAHFLLRKYRAKEL), MAGE-A4_134 − 160_ (MLP-2, AEMLERVIKNYKRCFPVIFGKASESLK), and MAGE-A4_274 − 306_(MLP-3, RALAETSYVKVLEHVVRVNARVRIAYPSLR) that have been derived from the sequence of the human tumor-specific antigen MAGE-A4.

**Fig. 2:**
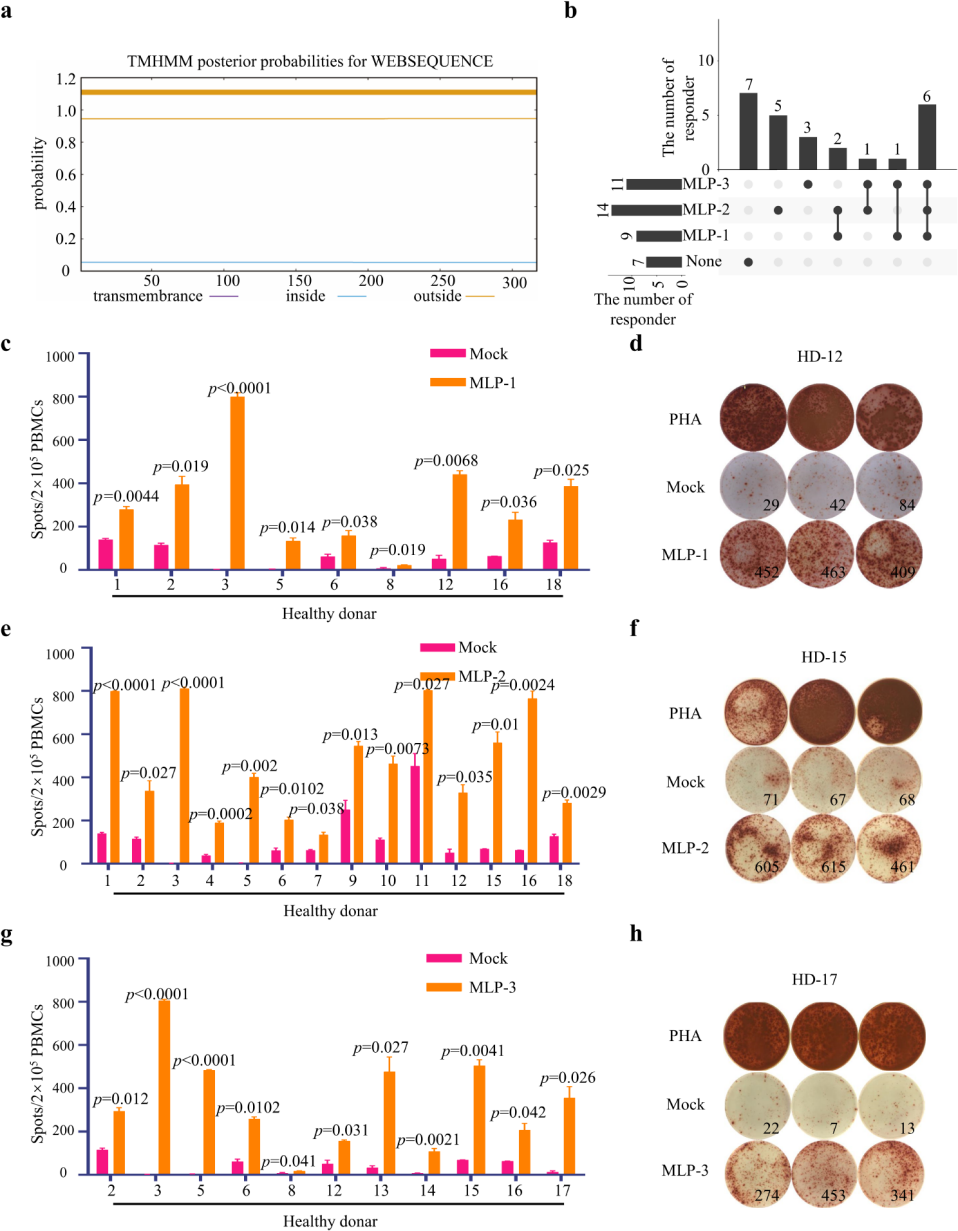
Immune responses of three candidate MLP were shown by ELISpot in healthy donors. **a**) TMHMM was predicted the location of intervened loop regions and the transmembrane helices in the protein topology, from 1to 317 residue (outside). **b**) The upset plot showed the number of immune responders in all 25 healthy donors. **c**) **e**) **g**) Histogram of IFN-γ secretion analysis. Healthy donors autologous PBMCs were stimulated with 3 candidate MAGE-A-4 long peptide (MLP-1, MLP-2, MLP-3) after which IFN-γ ELISpot were performed to assess the peptide-specific T cell responses respectively. no peptide media were used as negative control (Mock group). The IFN-γ secretion of peptide-stimulated PBMCs more than 2-fold Mock were considered to have positive immune responsethe, upper limit of detection was 800 spots per well. **d**) **f**) **h**) Representative images of positive immune response were produced by stimulation of PBMCs from three healthy donors with MLP-1, MLP-2, and MLP-3, respectively. Phytohemagglutinin (PHA) was used as the positive controls Data were shown as the mean ± SEM, P-values were calculated by unpaired Student’s t-tests (*n* = 3)

The amino acid sequence of MAGE-A4 contains 317 amino acids, which ectodomain residues ranging from 1 to 317, predicted using TMHMM (Fig. 2a). Based on a comprehensive survey of HLA Class I and HLA Class II restrictive locus, which incorporated a total of 812,211 individuals throughout 31 provinces, autonomous regions, and municipalities in China, we have chosen six HLA-A alleles, namely HLA-A*01:01, HLA-A*02:01, HLA-A*11:01, HLA-A*24:02, HLA-A*30:01, and HLA-A*33:03. These alleles represent 3.59%, 12.04%, 20.89%, 15.55%, 5.97%, and 8.23% of the Chinese population, respectively. In the meantime, we have opted to select 8 HLA-DRB1 allels, which encompass HLA-DRB1*07:01, HLA-DRB1*09:01, HLA-DRB1*11:01, HLA-DRB1*15:01, HLA-DRB1*04:05, HLA-DRB1*03:01, HLA-DRB1*08:03, and HLA-DRB1*12:02. These alleles constitute 9.66%, 14.79%, 5.63%, 11.58%, 4.82%, 5.1%, 6.31%, and 8.71% of the Chinese population, respectively [23]. (Supplementary Fig S1). We examined the amino acid sequence of MAGE-A4 to determine long peptide epitope comprising the most potential MHC class I and II binding epitopes through affinity sites epitope target prediction website. We have opted for the antigen sequences designated as MAGE-A4_102 − 130_ (MLP-1), MAGE-A4_134 − 160_ (MLP-2), and MAGE-A4_277 − 306_ (MLP-3). The water solubility of the three candidate MAGE-A4 long peptide sequences was deemed satisfactory, predicted using Pepcalc. MLP-1, MLP-2, MLP-3 contains multiple peptide motifs with high affinity for common and major MHC class I and class II molecules (Table 1).

**Table 1:** Potential epitopes for different MHC molecules contained in the MLP vaccine

LSP name	CD8 + T-cell epitope			CD4 + T-cell epitope		
	Position	Sequence	HLA class I restriction	Position	Sequence	HLA class II restriction
MLP-1: AESLFREALSNKVDELAHFLLRKYRAKEL						
	113–121	KVDELAHFL	A*01:01	116–130	ELAHFLLRKYRAKEL	DRB1*12:02
	105–113	LFREALSNK	A*30:01	102–115	AESLFREALSNKVD	DRB1*07:01
	104–113	SLFREALSNK	A*11:01	117–130	LAHFLLRKYRAKEL	DRB1*15:01
	109–117	ALSNKVDEL	A*02:01	104–117	SLFREALSNKVDEL	DRB1*04:05
	116–126	ELAHFLLRKYR	A*33:03	103–117	ESLFREALSNKVDEL	DRB1*08:03
MLP-2: AEMLERVIKNYKRCFPVIFGKASESLK						
	146–154	RCFPVIFGK	A*11:01	135–148	EMLERVIKNYKRCF	DRB1*11:01
	136–144	MLERVIKNY	A*01:01	141–154	IKNYKRCFPVIFGK	DRB1*09:01
	151–157	IFGKASESL	A*24:02	134–148	AEMLERVIKNYKRCF	DRB1*03:01
				138–151	ERVIKNYKRCFPVI	DRB1*15:01
				136–150	MLERVIKNYKRCFPV	DRB1*12:02
MLP-3: RALAETSYVKVLEHVVRVNARVRIAYPSLR						
	286–291	KVLEHV	A*02:01	288–302	LEHVVRVNARVRIAY	DRB1*08:03
	297–305	RVRIAYPSL	A*30:01	293–306	RVNARVRIAYPSLR	DRB1*03:01
	283–291	SYVKVLEHV	A*24:02	289–302	EHVVRVNARVRIAY	DRB1*12:02
	278–286	ALAETSYVK	A*11:01	282–296	TSYVKVLEHVVRVNA	DRB1*15:01
	284–293	YVKVLEHVVR	A*33:03			

### Induction of T-cell responses specific for MLPs in healthy donors

To evaluate the T-cell immune responses elicited by the three potential long peptides, we extracted PBMCs from 25 healthy donors with varying HLA-A alleles and stimulated them with each MLP peptide (Supplementary Table S1). We subsequently conducted IFN-γ-based ELISpot assays on the stimulated PBMCs for each MLP peptide. A total of 72% (18/25) of healthy individuals exhibited a specific T-cell response to the MLP peptide. Among the peptides tested, MLP-2 elicited the highest number of specific T-cell responses, observed in 56% (14/25) of the donors. Specific T-cell responses were also observed against MLP-1 and MLP-3, generating responses in 36% (9/25) and 44% (11/25) of the donors (Fig. 2b), respectively. The statistical analysis of the number of IFN-γ spots produced by different Chinese populations upon induce to each MAGE-A4 long peptide is illustrated through Fig. 2c and e, and 2g, and the absence of peptide stimulation is served as the negative control (Mock) in the study. Utilizing the ELISpot assays, stimulated PBMCs by the long peptides of MAGE-A4 were tested. The resulting data is illustrated in representative plots in Fig. 2d and f, and 2h. All ELISpot results are comprehensively displayed in Supplementary Fig S2. The data gathered collectively indicated that the MLP has a high level of T-cell immunogenicity in healthy individuals. In addition, it has been confirmed that the MLP has the potential to generate MAGE-4 specific T-cell responses through vaccination, which are not restricted to an individual’s HLA alleles.

### Cytotoxic T cell cytotoxicity assay

**Fig. 3:**
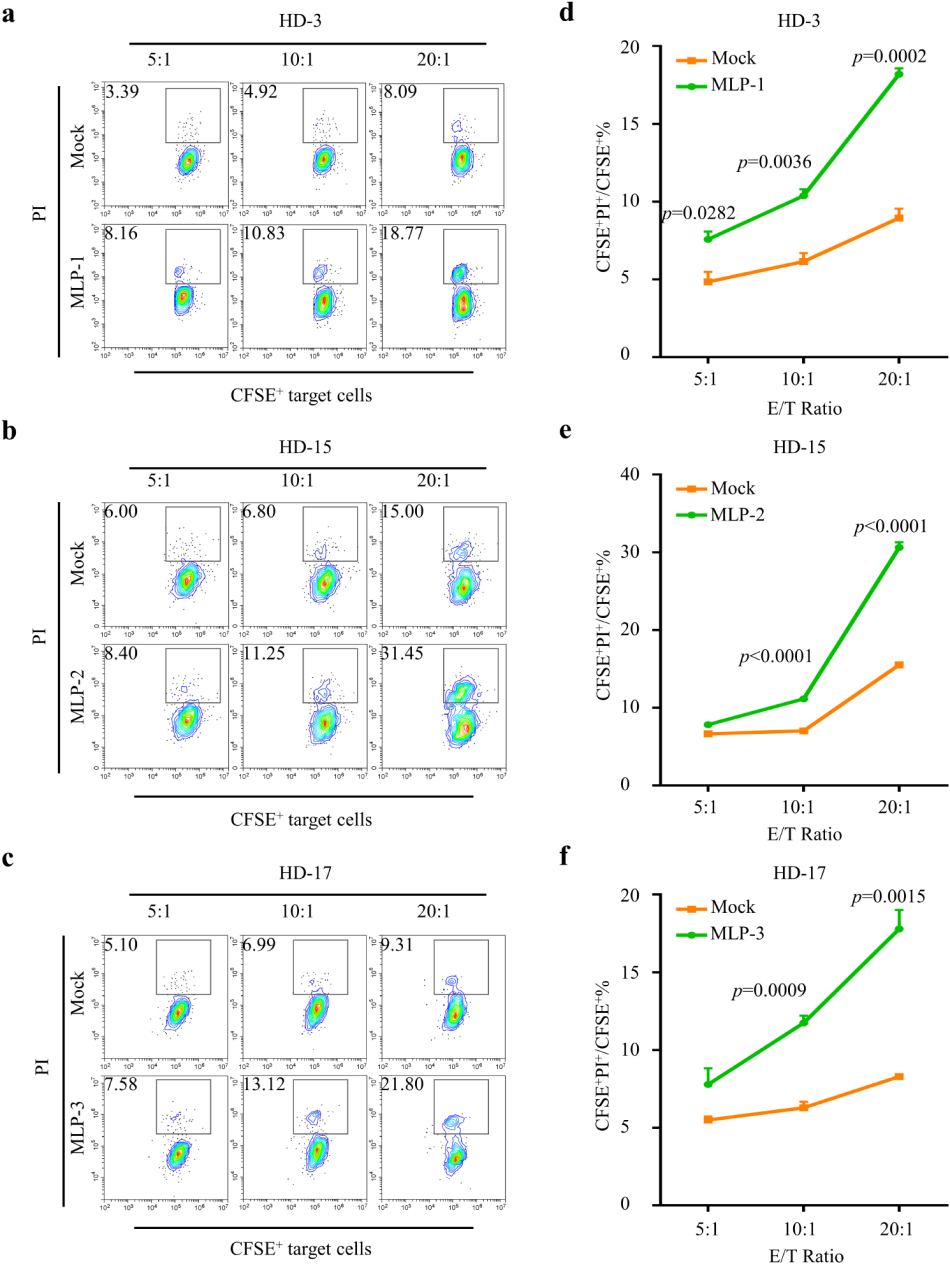
Cytotoxicity assay of peptide-specific T-cells induced by MLPs in vitro. Autogenous PBMCs from HD-3, 15, 17 were demonstrated a robust peptide-specific T cell responses when stimulated with MLP-1, MLP-2, MLP-3 conditions respectively. Cytotoxic T cell were incubated with CFSE labeled target cells at effector-to-target ratio (E: T) of 5:1, 10:1, 20:1. PI was added 10 h after incubation and the proportion of dead tumor cells(CFSE^+^PI^+^/CFSE^+^) was analyzed by flow cytometry. **a**) **b**) **c**) Representative flow cytometry images and **d**) **e**) **f**) The percentage of CFSE^+^PI^+^/PI^+^ cell population. The group without peptide induction served as the Mock group. Data were shown as the mean ± SEM, *P*-values were calculated by unpaired Student’s t-tests (*n* = 3)

Upon stimulation with MLP-1, MLP-2, and MLP-3, PBMCs derived from HD-3, HD15, and HD17, respectively, exhibited a strong and specific IFN-γ response to the antigens. In each peptide stimulated condition, the number of spots obtained was more than 10 times higher than the number obtained without peptide. The IFN-γ ELISpot assay demonstrated marked changes in peptide-specific IFN-γ secretion subsequent to MLP stimulation. To further define the function of cytotoxic T cells after MLP induced, the cytotoxicity of cytotoxic T cells was assessed using a CFSE/PI assay. PBMCs obtained from HD3, HD15, and HD17 were stimulated with MLP-1, MLP-2, and MLP-3, respectively, to induce the production of cytotoxic T cells. The generated cytotoxic T cells were then co-cultured with target cells labeled with CFSE at an effector to target ratio of 5:1, 10:1, and 20:1. As the effector-to-target ratio increased, the cytotoxic T cells displayed a remarkable enhancement in their ability to selectively eliminate their designated targets. We demonstrated that a 20:1 effector-to-target ratio resulted in a 2-fold increase in the percentage of deceased tumor cells across all peptide-stimulated conditions when compared to the Mock group (The group without peptide induction served as the Mock group) (*p* = 0.0002, *p* < 0.0001 and *p* = 0.0015, respectively, Fig. 3d and e and f). Furthermore, at a 10:1 effect-target ratio, each peptide exhibited significant statistical variation compared to the control group (*p* = 0.0036, *p* < 0.0001 and *p* = 0.0009, respectively). The evidence presented indicated that the PBMCs stimulated by the three long peptides being evaluated manifested significant cytotoxic effects. We chose distinct gastric cancer and melanoma cell lines as target cells, and both showed significant killing effects.

### Phenotypic characterization of vaccine-specific T cells by staining for surface markers and intracellular staining of cytokine

**Fig. 4:**
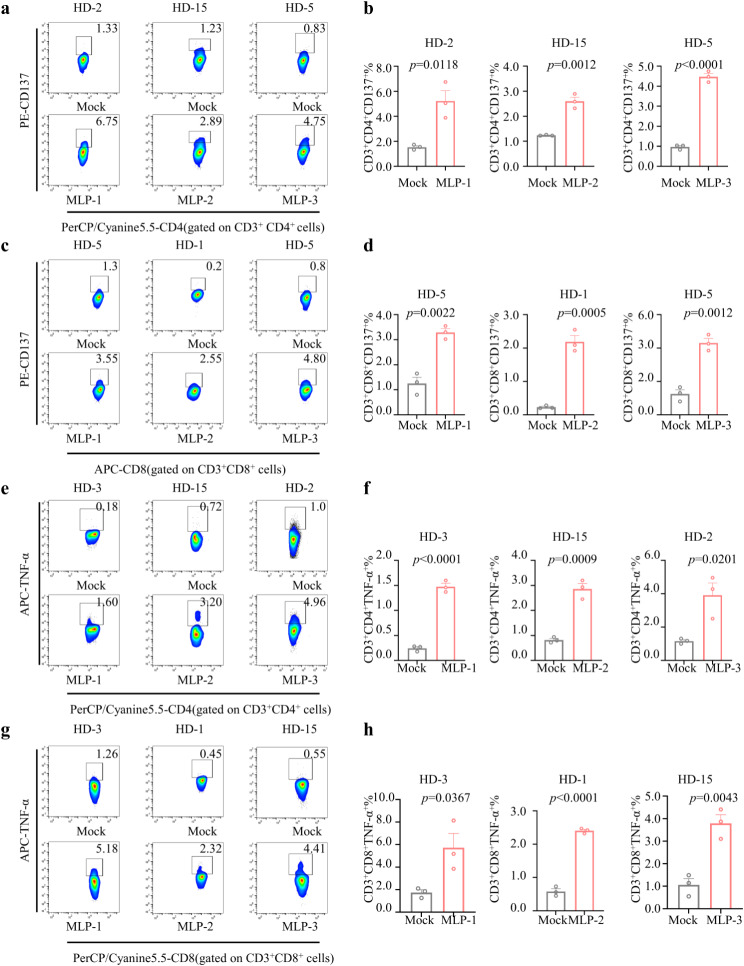
The multifunctional CD4^+^and CD8^+^T cell responses assay of T-cells induced by MAGE-A4 long peptides (MLPs). **a**) The representation flow cytometry images of the CD137 expression of peptide-induced CD4^+^ T cells in vitro measurement. **b**) the percentages of CD4^+^CD137^+^ T cells. The representation flow cytometry images of intracellular) The representation flow cytometry images of the CD137 expression of peptide-induced CD8^+^ T cells in vitro measurement. **d**) the percentages of CD8^+^CD137^+^ T cells. **e**) The representation flow cytometry images of intracellular TNF-α-positive cells among CD4^+^ T cells in vitro measurement. **f**) the percentages of intracellular CD4^+^TNF-α^+^T cells. (**g**) The representation flow cytometry images of intracellular TNF-α-positive cells among CD8^+^ T cells in vitro measurement. (**h**) the percentages of intracellular CD8^+^TNF-α^+^T cells. The group without peptide induction served as the Mock group. Data were shown as the mean ± SEM, *P*-values were calculated by unpaired Student’s t-tests (*n* = 3)

To further assess the function of antigen reactive T cells, we examined the percentage of CD137 and TNF-α produced by CD4^+^ and CD8^+^ T cells after MLP induced. PBMCs obtained from HD3, HD15, and HD17 were stimulated with MLP-1, MLP-2, and MLP-3, resulting in the generation of antigen-reactive T cells. The immunogenicity of these T cells was assessed using ELISpot. The proportion of CD3^+^CD4^+^CD137^+^ in the candidate peptide group was observed to be at least 2-fold higher than that of the Mock group (The group without peptide induction served as the Mock group) (Fig. 4a and b), the candidate peptide group displays a CD3^+^CD8^+^CD137^+^ cell proportion that is at least twice as high as that of the Mock group (Fig. 4c and d). In particular, the MLP-2 group exhibited a significantly higher proportion of CD3^+^CD8^+^CD137^+^ cells, which was approximately 10 times greater than that observed in the Mock group(*p* = 0.0005). The data presented in Fig. 4e and f for ICS indicates that the proportion of CD4^+^ T cells that produce TNF-α is greater in all groups that were induced by candidate peptides than in those that were not. Additionally, the candidate peptide induced a greater percentage of CD8^+^ T cells to produce TNF-α than the non-candidate peptide in all groups (Fig. 4g and h). In contrast to the Mock group, the MLP-1 and MLP-2 groups exhibited a significant increase of at least 2-fold in the proportion of CD3^+^CD4^+^ TNF-α^+^ and CD3^+^CD8^+^ TNF-α^+^ T cells.

### In vitro immunogenicity of MLPs and immunostimulatory of MLPs with R848 on DCs in mice

**Fig. 5:**
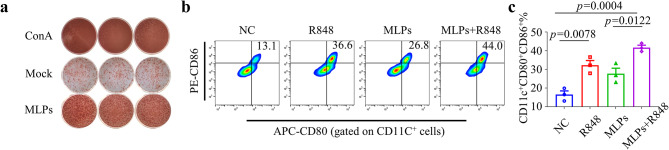
Immune responses of three candidate MAGE-A4 long peptides (MLPs) were shown in vitro in mice. **a**) The splenocytes isolated from Balb/C were stimulated with MLPs to assess the peptide-specific T-cells responses. IFN-γ ELISpot showed changes in peptide-specific IFN-γ secretion in vitro. no peptide stimulation and ConA represent the negative and positive control, respectively. **b**) The representation flow cytometry images of mature DCs (CD11c^+^CD80^+^CD86^+^) after co-incubation with R848, MLPs, or MLPs + R848 in vitro for 24 h. **c**) The percentage of CD11c^+^CD80^+^CD86^+^ cells. Data were shown as the mean ± SEM, *P*-values were calculated by unpaired Student’s t-tests(*n* = 3)

Our investigation involved the utilization of the prediction tool IEDB(http://tools.iedb.org/mhci/) to determine whether MLP possesses a significant affinity for Balb/c MHC class I and II (Table 2). To assess the immunogenicity of MLP in animals, a co-culture system of DCs and T cells was utilized to stimulate the production of specific T cells from the splenocytes of Balb/c mice. The MLP mixture group displayed the most pronounced response, as per the results, the Mock group (The group without peptide induction served as the Mock group), ConA as positive control (Fig. 5a). Toll-like receptor (TLR) 7/8 agonist imiquimod can stimulate the activation of antigen-presenting cells (APCs). Hence, we have formulated a cancer peptide vaccine that comprises R848 and MLP. To validate the capacity of R848 to enhance DC activation of the MLP vaccine, we conducted an incubation of the MLP and R848 combination with BMDCs, and subsequently assessed the levels of CD80 and CD86 expression on the BMDCs via flow cytometry. PBS was used as negative controls. The activation impact of the combination on BMDCs exhibited a significant enhancement of up to 43.5%. In comparison with MLP alone, the percentage of activated DCs increased by 11.3% (p = 0.0122), which suggests that R848 may serve as a promising adjuvant to stimulate the maturity of DCs in cancer peptide vaccines (Fig. 5b and c).

**Table 2:** Potential epitopes for different MHC molecules contained in the MLP vaccine in mice

LSP name	CD8 + T-cell epitope			CD4 + T-cell epitope		
	Position	Sequence	MHC I restriction	Position	Sequence	MHC II restriction
MLP-1: AESLFREALSNKVDELAHFLLRKYRAKEL						
	113–121	KVDELAHFL	H-2-Kd	116–129	ELAHFLLRKYRAKE	H2-IEd
	102–110	AESLFREAL	H-2-Ld	102–114	AESLFREALSNKV	H2-IAb
				119–130	HFLLRKYRAKEL	H2-IEd
MLP-2: AEMLERVIKNYKRCFPVIFGKASESLK						
	142–150	KNYKRCFPV	H-2-Dd	134–148	AEMLERVIKNYKRCF	H2-IEd
	151–159	IFGKASESL	H-2-Kd			
MLP-3: RALAETSYVKVLEHVVRVNARVRIAYPSLR						
	297–305	RVRIAYPSL	H-2-Ld	292–306	VRVNARVRIAYPSLR	H2-IAd
	283–291	SYVKVLEHV	H-2-Kd	289–302	EHVVRVNARVRIAY	H2-IEd
	286–294	KVLEHVVRV	H-2-Dd			

### In vivo immunization assay

**Fig. 6:**
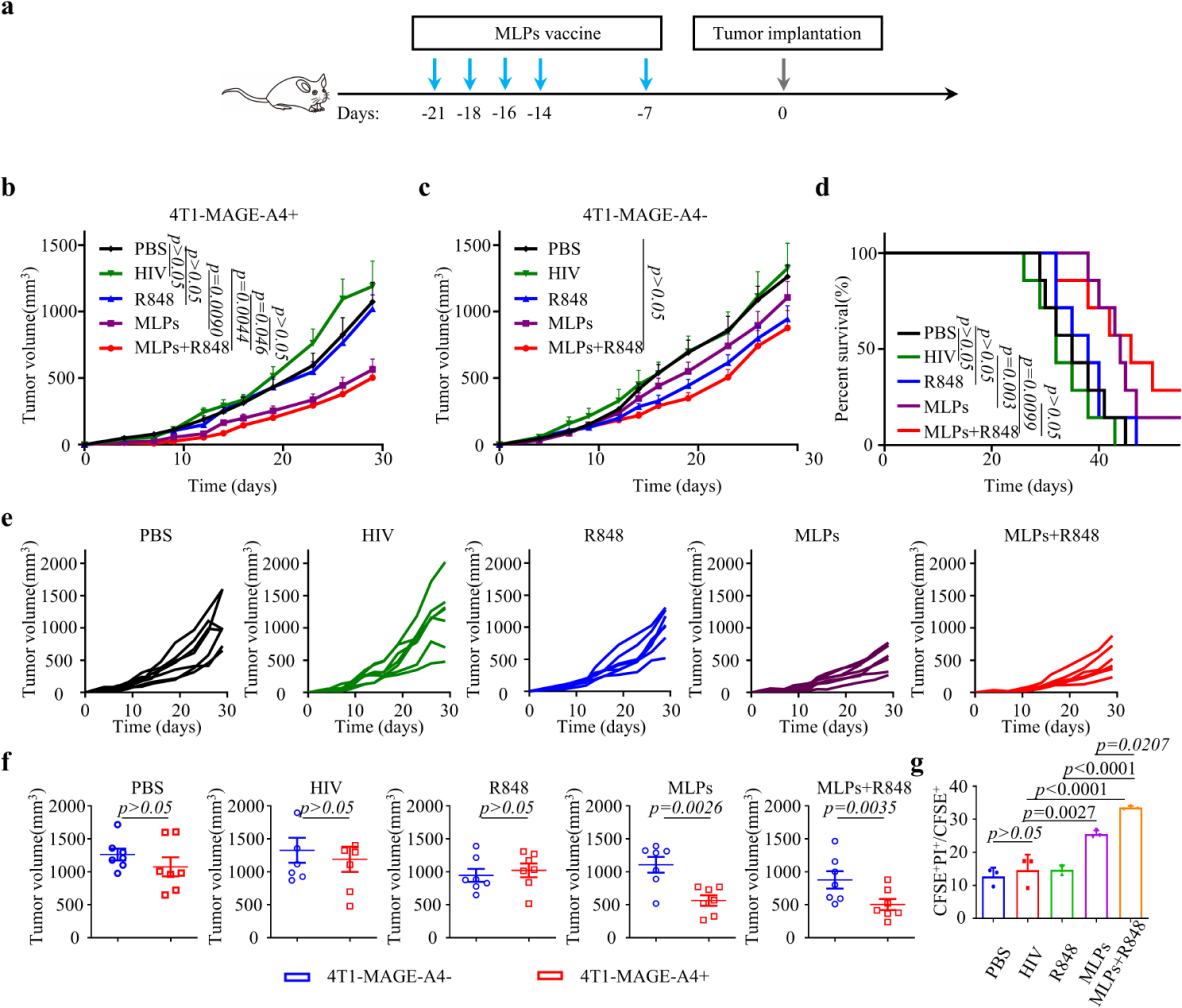
In vivo antitumor effect of MLPs vaccine. **a**) Schematic diagram of administration route for tumor immunotherapy in Balb/C mice with MLPs vaccines. 3 × 10^5^ 4T1-MAGE-A4 cells in 50µL PBS were injected subcutaneously on the right abdomen and 4T1 cells were inoculated on the left abdomen with same method and condition. Average tumor-growth curves of Balb/C mice bearing. **b**) 4T1-MAGE-A4 ^+^. **c**) 4T1 breast tumor with different treatments as indicated (*n* = 7). ( PBS group: 100µL PBS; HIV group: 60 µg HIV long peptide; R848 group: 10 µg R848; MLPs group: 60 µg MLP-1, 60 µg MLP-2, 60 µg MLP-3 mixture; MLPs + R848 group: 60 µg MLP-1, 60 µg MLP-2, 60 µg MLP-3, 10 µg R848 mixture). HIV long peptide as irrelevant peptide (*n* = 7). **d**) Survival curves of Balb/C mice in different groups (*n* = 7). **E**) Growth of 4T1-MAGE-A4^+^ tumors in mice immunized with different vaccine formulation. **f**) Growth of 4T1-MAGE-A4^+^ and 4T1 tumors in mice immunized with different vaccine formulation on day 29. **g**) Cytotoxicity assay of peptide-specific T-cells induced by MLPs in vivo. When the mice were immuned after vaccination, lymphocytes in spleens were incubated with CFSE labeled 4T1-MAGE-A4^+^ breast cancer cells at effector-to-target ratio (E: T) of 10:1. PI was added 10 h after incubation and the proportion of dead tumor cells (CFSE^+^PI^+^/CFSE^+^) was analyzed by flow cytometry(*n* = 3). Data were shown as the mean ± SEM, *P*-values were calculated by unpaired Student’s t-tests (*n* = 3)

The establishment of a subcutaneous two-tumor immunoprevention mouse model in female Balb/c mice was carried out to determine the immunopreventive efficacy of the cancer peptide vaccine and its antigen specificity towards MAGE-A4. (Fig. 6a). Obviously, the bilateral tumors of mice grew rapidly in the PBS group, the HIV group and R848 group. The MLP vaccine showed the best tumor growth inhibition (Fig. 6b and c) and was associated with prolonged survival in mice (Fig. 6d). The growth rate of tumors on the left side was significantly slower in both the MLP and MLP + R848 groups, whereas the growth rate of tumors on the right side did not differ significantly from the other groups. Tumor size exhibited a considerable dissimilarity on both sides among the MLP group and the MLP + R848 group. Conversely, the differences in bilateral tumors among the PBS, HIV, and R848 groups were minimal. it revealed that the tumor vaccine exhibited specificity. The 29th day following tumor inoculation revealed distinct presentations of bilateral tumors within different groups, a marked variation in bilateral tumor volume was also significant different between the MLP group and the MLP + R848 group (Fig. 6f). Upon extraction of spleen cells from immunized mice, in vitro incubation with 4T1-MAGE-A4^+^ cells were conducted. The killing capacity of MLP group and MLP + R848 group was observed to be markedly distinct from that of the unrelated peptide group at an effect-target ratio of 10:1. Additionally, the MLP + R848 group exhibited a superior capacity for inducing cytotoxicity compared to the MLP group, thus implying that R848 served as a beneficial adjunct in the vaccine. (Fig. 6g)

Following the administration of the vaccine, the mice were humanely euthanized seven days later and their inguinal lymph nodes and spleens were collected to assess the expression of surface markers related to memory T cells through flow cytometry.

**Fig. 7:**
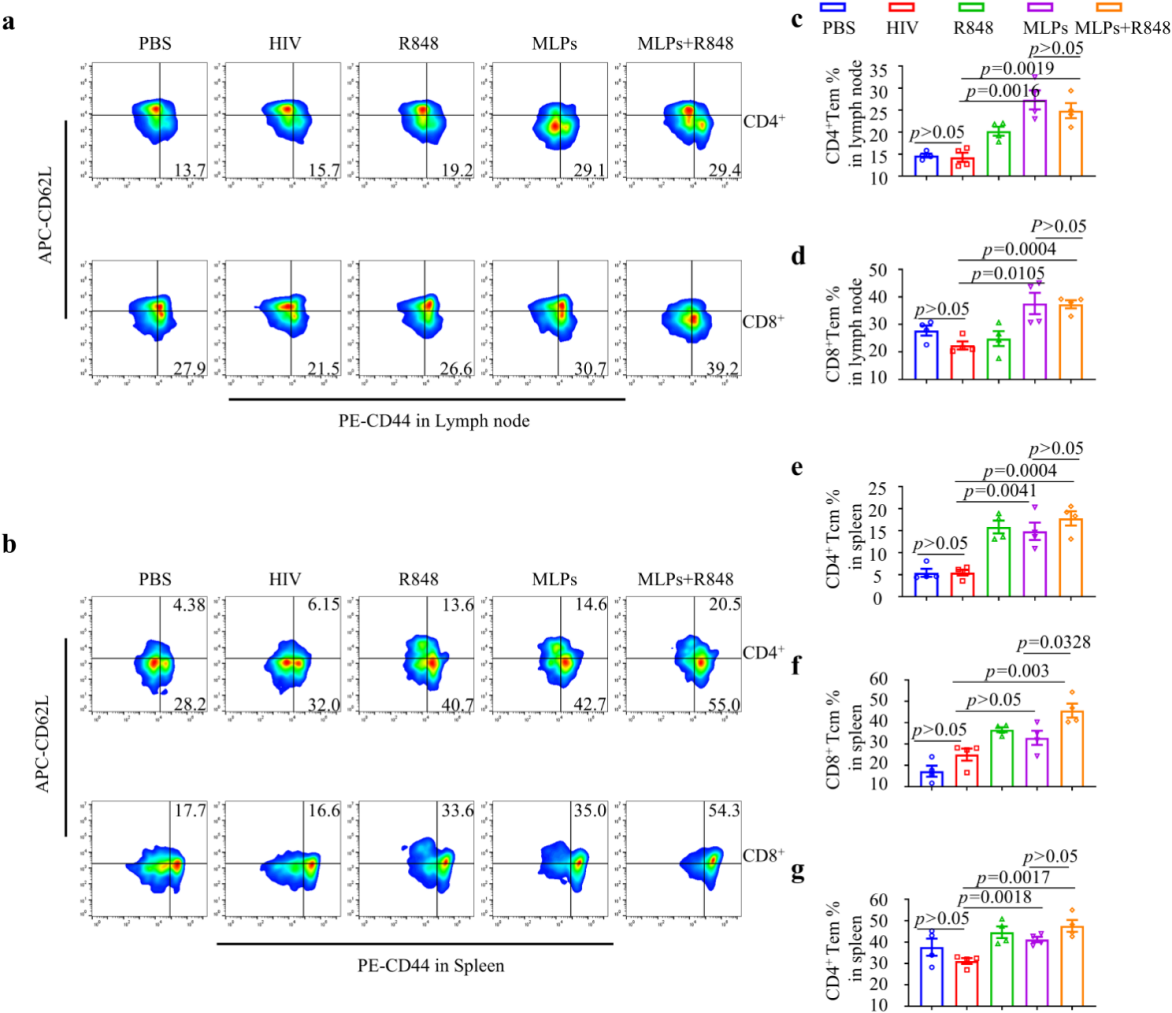
T cell immune response induced by MLPs vaccine. Balb/C mice was immunized in different group, 2 weeks after the last administration, Spleens, lymph nodes were collected and analyzed. the representation flow cytometry images of central memory T cell (Tcm) and effector memory T cell (Tem) in CD4^+^ or CD8^+^ T cells respectively in in **a**) Lymph nodes, **b**) Spleens. Percentage of Tcm in Lymph nodes in **c**) CD4^+^T cells and **d**) CD8^+^ T cells. Percentage of Tem in spleen **e**) CD4^+^ T cells **f**) CD8^+^ T cells. **g**) Percentage of Tcm in CD8^+^ T cells in spleen. Data were shown as the mean ± SEM, *P*-values were calculated by unpaired Student’s t-tests

Within lymph nodes, the MLP and MLP + R848 groups displayed a greater prevalence of effector memory CD8^+^ T (Tem, CD8^+^CD44^+^CD62L^−^) cells, with mean proportions of 37.63% and 37.32%, respectively. In contrast, the PBS and HIV groups exhibited lower percentages of CD8^+^ Tem cells, measuring at 27.78% and 22.4%, respectively. The trend was similarly observed for CD4^+^ Tem cells in lymph nodes (Fig. 7a, c and d). The spleen analysis revealed that the MLP alone and MLP + R848 groups had a significantly higher proportion of CD8^+^ central memory T cells (Tcm, CD8^+^CD44^+^CD62L^+^) cells, at 32.88% and 45.6%, respectively, compared to the PBS (17.225%) and HIV (25.03%) groups. This trend was also observed in the percentage of CD4^+^ Tcm cells. Additionally, it was observed that the population of CD4^+^ Tem cells in the spleen exhibited a marked increase in the MLP (41.18%) and MLP + R848 (47.6%) groups in contrast to the HIV (31.1%) group (Fig. 7b, e, f and g). This suggests that the screened long peptides may have the properties of adjuvant peptides, and these findings also suggest the possibility of immune effector activity and anti-tumor response.

### Biosafety assessment

The crucial factor of biological safety was given due consideration in the implementation of the MLP vaccine. The H&E stained images of the major organs extracted on the 7 days subsequent to the final vaccine administration, as presented in Supplementary Fig S3a, demonstrated no evident damage in any of the groups. Moreover, the weight of each mouse was increased, the trend of body weight change remained stable during treatment in all groups, there was no significant difference. (Supplementary Fig S3b). Furthermore, we observed if there was systemic inflammation and evaluated the hepatic and renal function of Balb/c mice in five distinct groups, including PBS, HIV, R848, MLP, and MLP + R848. It is apparent that the levels of aspartate aminotransferase (AST), alanine aminotransferase (ALT), alkaline phosphatase (ALP), blood urine nitrogen (BUN), and creatinine (CREA) among the five groups were generally consistent and within normal limits (Supplementary Fig S3c, S3d). Therefore, the MLP vaccine was examined with good biological safety.

## Discussion

The tumor-associated antigen MAGE-A4 exhibits a notably elevated expression in various types of tumors. MAGE-A4 is present in notable levels in triple negative breast cancer. [28–30] After conducting an allele analysis of 812,211 volunteer donors from the Chinese Bone Marrow Donation Program (CMDP), we identified alleles with elevated frequencies at the HLA-A and HLA-DRB1 loci within the population. Simultaneously, the choice of alleles was predicated upon the principles and attributes of the HLA supertype [31, 32]. The selection process involves opting for a supertype that exhibits 1–2 alleles (Supplementary Table S2). After careful consideration, a total of 6 HLA-I and 8 HLA-II subtypes were ultimately chosen, as they were found to provide coverage for 66.26% and 66.6% of the Chinese population, respectively. Based on the HLA supertypes identified, it is possible to encompass 86.14% and 76.1% of the entire populace in China, respectively (Supplementary Fig S1b).

It was shown that the administration of three multi-epitope peptides of MLP can elicit robust immune responses in the peripheral blood mononuclear cells (PBMC) of various subtypes of healthy individuals in China. CD137 and TNF-α were observed and verified to be expressed in IFN-γ-T cells with immunoreactivity, which promote the differentiation of T cell memory and effector factors, and provide T cells with protection against apoptosis [33]. When IFN-γ positive T-cells, previously demonstrated to be immunoreactive, were subjected to in vitro incubation with target cells, the outcome revealed that CTLS, which were specific to peptide and induced by three MAGE-A4 multi-epitope peptides, were capable of recognizing and eliminating target cells, the long peptide we screened had a pan-tumor killing effect, which was universally applicable. These CTLs demonstrated robust killing ability and immune function.

In formulating the MAGE-A4 long peptide sequence, our approach commenced with the meticulous screening of MHC-I short peptides featuring high affinity (9–11 amino acids). Subsequently, we amalgamated and expanded various short peptide epitopes to formulate extended long peptide sequences possessing multiple MHC affinities. A pivotal aspect of this methodology lies in ensuring that upon in vivo inoculation, the short peptide epitopes encapsulated within the long peptide sequences retain their original MHC affinity, thereby eliciting specific T cell responses. To provide experimental evidence of this aspect in vivo, we employed Balb/c mice inoculated with 4T1 cells transfected with human MAGE-A4 to establish a breast cancer model.

Notably, the selection of MAGE-A4 sequences was predicated on human MHC affinities. While acknowledging the dissimilarity between human and murine MAGE-A4 proteins, our investigation revealed that the MHC of Balb/c mice (H-2Kd), harbored high-affinity epitopes within the selected sequences (Table 2). Consequently, we did not directly use cell lines expressing murine MAGE-A4, but transfected human MAGE-A4 into murine 4T1 breast cancer cells. This design confirms the key aspect mentioned above and underscores a perspective on clinical applicability: In cancer patients expressing either tumor neoantigens or cancer-testis antigens, the administration of long peptide vaccination elicits advantageous immune responses, contingent upon the inclusion of their MHC high-affinity epitope within the long peptide, irrespective of its provenance from the original protein.

The potential for clinical transformation of the vaccine was evaluated by developing a mouse model that prevented double tumors in triple negative breast cancer. The efficacy and safety of the long peptide vaccine were then established through rigorous testing. It has been ascertained that the vaccine possesses the capacity to specifically hinder the growth of tumors that display high expression of 4T1-MAGE-A4^+^. The utilization of flow cytometry has revealed a significant upsurge in the levels of effector memory CD4^+^ T and CD8^+^ T cells in the lymph node cells of mice that were subjected to the vaccine. Similarly, the spleen cells of these mice exhibited an increase in effector memory CD4^+^ T cells. Additionally, there is a significant elevation in the levels of central memory CD4^+^ T and CD8^+^ T cells, which have the potential to differentiate into effector CD4^+^ T and CD8^+^ T cells and exert anti-tumor effects. It appears that the MLP could be a potential adjuvant peptide, based on these results. R848 leads to the activation of antigen presentation and the polarization of APC and T cell responses as TLR7/8 immunomodulator [34]. Upon being injected subcutaneously, it triggers the maturation of DCs and subsequently heightens the efficacy of antigen-specific cytotoxic T lymphocytes (CTLs). The enhancement of DC cell maturity in lymph node and spleen tissues of mice subjected to vaccination was not observed in our study. This may be attributed to the prompt metabolism and premature activation of minute molecules, which impeded the timely monitoring of the subject matter. The antitumor effect of the long peptide group was observed to a certain extent; however, the addition of R848 to the candidate peptide did not enhance the antitumor effect. One possible explanation for this phenomenon is that R848, being a small-molecule compound, undergoes rapid metabolism and has a brief retention period, thereby failing to elicit synergistic effects with long peptide cancer vaccine. To overcome the vaccine’s lack of immunogenicity, limited APC uptake, and inadequate targeting of lymphoid tissues. We hypothesize that tumor antigens can be attached to polymeric materials, with adjuvant components modified, through either covalent or non-covalent bonding. This delivery system affords not only the advantage of flexible antigen modification, but also the ability to manage loading efficiency. The aim is to increase immunogenicity and vaccine efficacy by targeting DCs to promote antigen internalization, activating and maturing DCs, and enhancing antigen cross-presentation and nanoparticle adjuvantability. The development of effective delivery systems for antigens and immune adjuvants is a significant research pursuit that aims to improve the clinical effectiveness of vaccines.

Currently available immunotherapy methods include Car-T cell therapy, TCR-T cell therapy, neoantigen strategies, etc. However, these approaches are time-consuming and costly while not fully meeting the clinical needs of patients. The value of utilizing long-peptide vaccines in clinical applications lies in their “off-the-shelf” feature. they can be administered based on patient HLA type and antigen expression before Car-T cell therapy or TCR-T cell therapy initiation or neoantigen vaccine administration. During this process, chemotherapy or radiotherapy can provide additional treatment time for patients and prolong their survival.

## Conclusions

A novel multi-epitope long peptide vaccine MLP was created, containing MHC I and MHC II restriction peptides that target human MAGE-A 4 antigen, and it was found to induce multi-epitope specific CTLs that are restricted to the HLA-A1, HLA-A2, HLA-A3, and HLA-A24 superfamily. We found that MLP successfully induced specific CTL responses in individuals expressing several common HLA alleles. The MLP vaccine, which has an inhibitory effect on mouse tumors, may be further enhanced when paired with an adjuvant such as R848 in the murine prevention model. This combination has the potential to increase the impact of immunotherapy in the mouse model. To sum up, our meticulously devised strategy yielded affirmative experimental outcomes, thereby affirming the broad applicability and efficacy of long peptide vaccination.

### Electronic supplementary material

Below is the link to the electronic supplementary material.

Supplementary Material 1

Supplementary Material 2

Supplementary Material 3

Supplementary Material 4

## Data Availability

No datasets were generated or analysed during the current study.

## Declarations

All animal experiments referring to animals were performed in accordance with the Guide and Care and Use of Laboratory Animals from the National Institutes of Health and ARRIVE. The animal testing was carried out in adherence to the guidelines laid down by the Laboratory Animal Care and Use Committee of the Affiliated Nanjing Drum Tower Hospital of Nanjing University Medical School (2021AE01084).

## Abbreviations

APCs: Antigen presenting cells
BMDCs: Bone marrow-derived dendritic cells
CMDP: Chinese bone marrow donation program
CFSE: Carboxy fluorescein succinimidyl amino ester
CTL: Cytotoxic T lymphocyte
DCs: Dendritic cells
HLA: Human leukocyte antigen
H&E: Hematoxylin and eosin
IFN-γ: Interferonγ
MHC: Major histocompatibility complex
PBMC: Peripheral blood mononuclear cells
TLR: Toll-like receptors
TNF-α: Tumor necrosis factor-alpha
