# Supplementary material for: Efficacy of MAGE-A4 long peptide as a universal immunoprevention cancer vaccine
Source: Cancer Cell Int. 2024 Jul 3;24:232. doi: 10.1186/s12935-024-03421-2 (PMC11223347; doi:10.1186/s12935-024-03421-2)
Supplement: Supplementary file 1 — Supplementary Material 1 [file 12935_2024_3421_MOESM1_ESM.docx]

**Table S1.** HLA typing of blood from all healthy donors.

| Healthy donors | HLA-A Genotype | |
| --- | --- | --- |
| HD-1 | HLA-A*02:03 | HLA-A*30:01 |
| HD-2 | HLA-A*02:01 | HLA-A*11:01 |
| HD-3 | HLA-A*02:01 | HLA-A*02:01 |
| HD-4 | HLA-A*03:01 | HLA-A*31:01 |
| HD-5 | HLA-A*11:01 | HLA-A*31:01 |
| HD-6 | HLA-A*24:20 | HLA-A*33:03 |
| HD-7 | HLA-A*02:01 | HLA-A*24:02 |
| HD-8 | HLA-A*02:01 | HLA-A*11:02 |
| HD-9 | HLA-A*02:01 | HLA-A*24:02 |
| HD-10 | HLA-A*01:01 | HLA-A*02:07 |
| HD-11 | HLA-A*02:01 | HLA-A*26:01 |
| HD-12 | HLA-A*31:01 | HLA-A*31:17 |
| HD-13 | HLA-A*02:01 | HLA-A*30:01 |
| HD-14 | HLA-A*02:07 | HLA-A*31:01 |
| HD-15 | HLA-A*02:01 | HLA-A*33:03 |
| HD-16 | HLA-A*26:01 | HLA-A*31:01 |
| HD-17 | HLA-A*11:01 | HLA-A*24:02 |
| HD-18 | HLA-A*11:01 | HLA-A*31:01 |
| HD-19 | HLA-A*26:01 | HLA-A*29:01 |
| HD-20 | HLA-A*11:01 | HLA-A*33:03 |
| HD-21 | HLA-A*02:01 | HLA-A*30:01 |
| HD-22 | HLA-A*24:02 | HLA-A*33:03 |
| HD-23 | HLA-A*02:06 | HLA-A*24:02 |
| HD-24 | HLA-A*02:06 | HLA-A*30:01 |
| HD-25 | HLA-A*02:01 | HLA-A*33:03 |

**Table S2** HLA class I and HLA Class II supertype classification and included genotypes.

| Classification of HLA | Supertype | Alleles |
| --- | --- | --- |
| HLA class Ⅰ | A1 | HLA-A*01:01, A*26:01, A*26:02 |
|  | A2 | HLA-A*02:01, A*02:02, A*02:03, A*02:04, A*02:05, A*02:06, A*02:07, A*68:02, A*69:01 |
|  | A3 | HLA-A*03:01, A*11:01, A*31:01, A*33:01, A*68:01 |
|  | A24 | HLA-A*24:02, A*30:01, A*23:01, A*24:03, A*24:04, A*30:02, A*30:03 |
| HLA class Ⅱ | Main DR | HLA-DRB1*01:01, DRB1*07:01, DRB1*09:01, DRB1*11:01, DRB1*12:01, DRB1*15:01 |
|  | DR4 | HLA-DRB1*04:01, DRB1*04:05, DRB1*08:02 |
|  | DRB3 | HLA-DRB1*03:01, DRB1*13:02 |
|  | Other | HLA-DRB1*08:03, DRB1*12:02 |
