# Supplementary figures and images for: Efficacy of MAGE-A4 long peptide as a universal immunoprevention cancer vaccine
Source: Cancer Cell Int. 2024 Jul 3;24:232. doi: 10.1186/s12935-024-03421-2 (PMC11223347; doi:10.1186/s12935-024-03421-2)

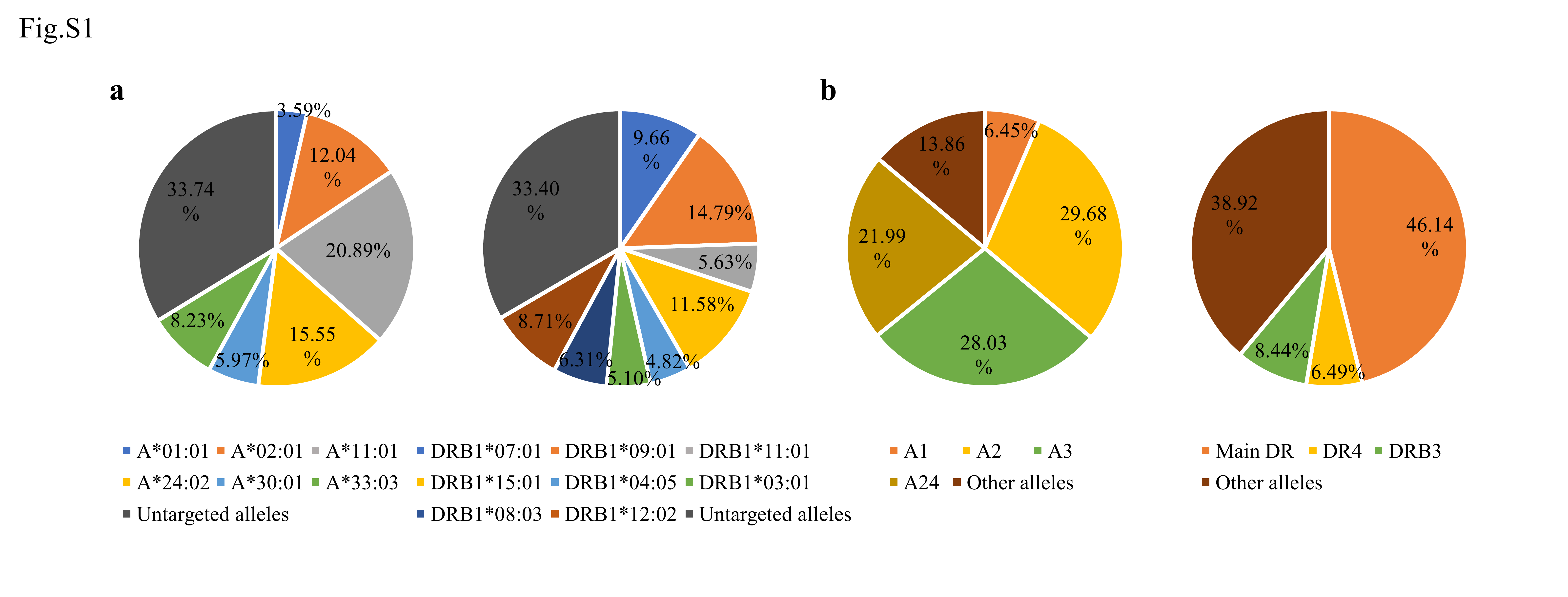

Supplement: Supplementary file 2 — Supplementary Material 2 [file 12935_2024_3421_MOESM2_ESM.tif]

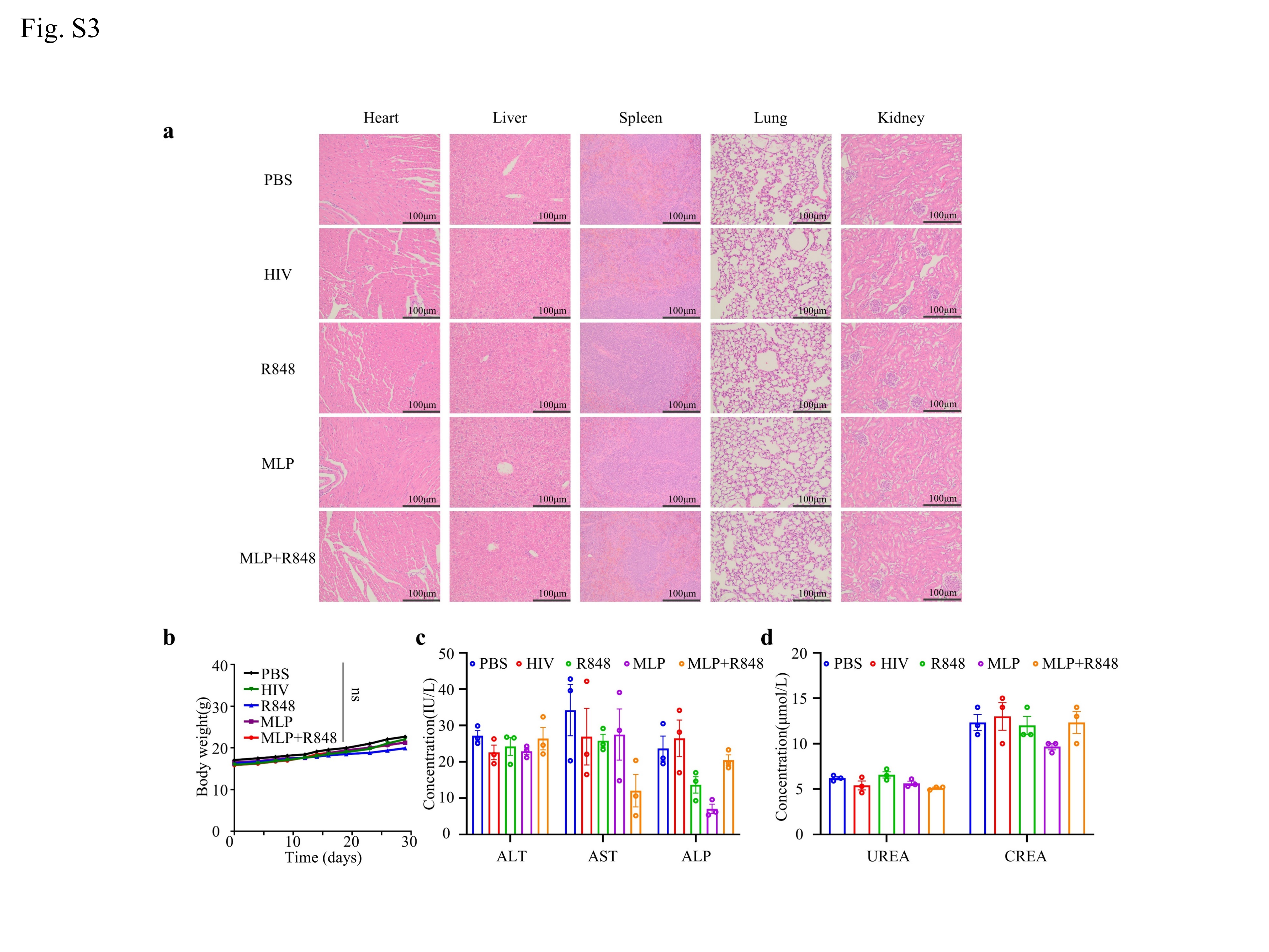

Supplement: Supplementary file 3 — Supplementary Material 3 [file 12935_2024_3421_MOESM3_ESM.tif]

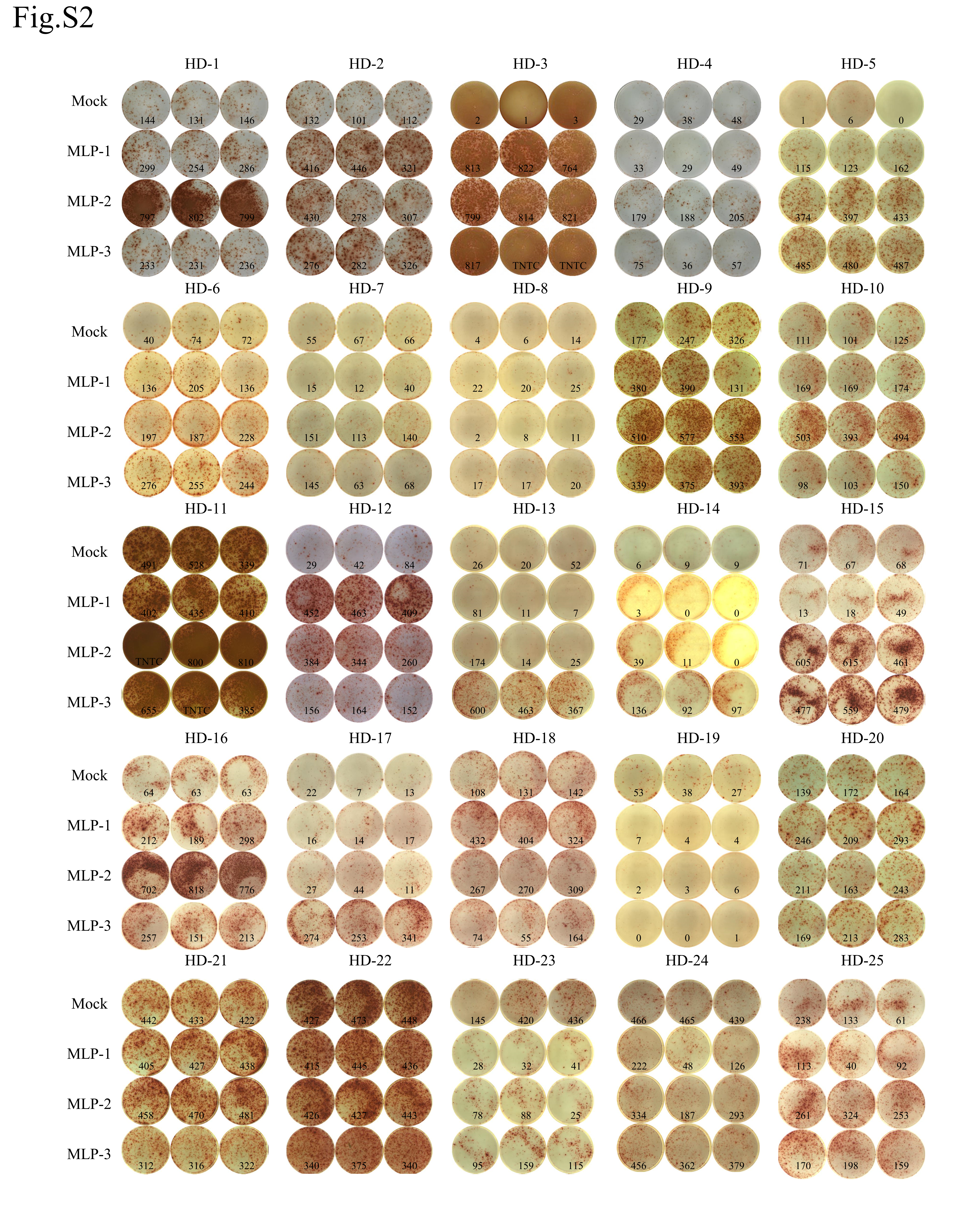

Supplement: Supplementary file 4 — Supplementary Material 4 [file 12935_2024_3421_MOESM4_ESM.tif]
